# Phase II trial of irinotecan plus docetaxel in cisplatin-pretreated relapsed or refractory oesophageal cancer

**DOI:** 10.1038/sj.bjc.6601168

**Published:** 2003-08-12

**Authors:** F Lordick, C von Schilling, H Bernhard, M Hennig, R Bredenkamp, C Peschel

**Affiliations:** 1 3rd Department of Internal Medicine (Haematology/Medical Oncology), Klinikum rechts der Isar, Technical University of Munich, Ismaninger Str. 22, D-81675 Munich, Germany; 2 Institute for Medical Statistics and Epidemiology, Technical University of Munich, Ismaninger Str. 22, D-81675 Munich, Germany; 3 Munich Center for Clinical Studies, Klinikum rechts der Isar, Ismaninger Str. 22, D-81675 Munich, Germany

**Keywords:** irinotecan, docetaxel, oesophageal cancer

## Abstract

This phase II trial assessed the toxicity and efficacy of irinotecan plus docetaxel in cisplatin-pretreated oesophageal cancer. Irinotecan 160 mg m^−2^ plus docetaxel 65 mg m^−2^ once every 3 weeks led to severe myelosuppression in four patients, all of whom experienced neutropenic fever. After amendment of this regimen, 24 patients (male/female=18/6; median age=58.5 years; ECOG performance status 0/1/2=9/11/4) with advanced oesophageal cancer (adenocarcinoma/epidermoid carcinoma=13/11) received irinotecan 55 mg m^−2^ plus docetaxel 25 mg m^−2^ on days 1, 8 and 15 of a 28-day cycle. Serious adverse events occurred in five patients, one with lethal outcome (pneumonia). Haematological toxicity ⩾3° was rare, whereas nonhaematological toxicity ⩾3° was noted in nine out of 24 patients (asthenia in five patients, diarrhoea in three patients, nausea/emesis in two patients, constipation in one patient). Median survival time was 26 (range 2–70) weeks. Response rate, assessed according to the WHO criteria, was 12.5% (95% CI 2.7–32.4%); rate of disease stabilisation (partial remission and stable disease) was 33.3% (95% CI 15.6–55.3%) with a median duration of 18.5 (range 16–51) weeks. Although the nonhaematological toxicity proved to be considerable, weekly irinotecan plus docetaxel is feasible and shows some activity in extensively pretreated patients with oesophageal cancer.

Owing to early lymphogenic and haematogenous spread, most patients with oesophageal cancer are diagnosed with advanced disease. These patients receive multimodal treatment if the tumour is deemed to be resectable, or systemic chemotherapy if palliation is the goal. Cisplatin has been the mainstay of chemotherapy and chemoradiotherapy protocols during the last decade (Herskovic *et al*, 1992; Bleiberg *et al*, 1997). In case of resistance to cisplatin-based therapy, no evidence-based recommendations regarding second-line treatment exist, but many patients ask for more than measures of best supportive care. Thus, there is a need for tolerable and active protocols beyond cisplatin-based therapy. Irinotecan and taxanes proved to induce remissions in oesophageal cancer (Ilson *et al*, 1998,1999) although single-agent docetaxel revealed only moderate activity (Heath *et al*, 2002). The combination of irinotecan and docetaxel has been investigated in phase I studies in patients with pretreated solid tumours (Adjei *et al*, 2000; Couteau *et al*, 2000). The recommended phase II dose on this schedule was irinotecan 160 mg m^−2^ followed by docetaxel 65 mg m^−2^ administered every 3 weeks (Adjei *et al*, 2000). This phase II trial was designed to assess the activity and toxicity of the combination of irinotecan plus docetaxel in cisplatin-pretreated relapsed or refractory oesophageal cancer.

## METHODS

### Patient population

Patients with histologically confirmed adenocarcinoma or epidermoid carcinoma of the oesophagus were enrolled at a single site (Klinikum rechts der Isar, Technical University of Munich). Eligible patients with metastatic disease or locally advanced disease not curable with surgery or radiation therapy were previously treated with cisplatin-based chemotherapy or chemoradiotherapy. Patients were greater than 18 years of age with an ECOG performance status ⩽2.

Adequate bone marrow, renal and hepatic function was necessary and was defined as an absolute neutrophil count (ANC) ⩾1500 *μ*l^−1^, platelets ⩾100 000 *μ*l^−1^, *S*-creatinine <1.5 × upper limit of normal (ULN), total bilirubin ⩽1.5 × ULN, SGOT and/or SGPT ⩽1.5 × ULN (⩽5.0 × ULN in the presence of liver metastasis). Patients were not allowed to have any CNS metastases, neuropathy ⩾grade 2 and had to have a life expectancy of ⩾12 weeks.

Pretreatment evaluation included signed written informed consent, complete history and physical examination, laboratory tests, CT scans of all tumour involved areas, ECG and urine or serum beta-HCG if the patient was a female of child-bearing age. The study was approved by the ethics committee for human research at the Technical University of Munich.

### Treatment plan

Initially, irinotecan was administered at a dose of 160 mg m^−2^ over 90 min immediately followed by docetaxel 65 mg m^−2^ over 60 min once every 3 weeks. One treatment every 3 weeks was considered one cycle. This regimen was amended after the treatment of four patients.

After the amendment, irinotecan was administered at a dose of 55 mg m^−2^ over 90 min followed by docetaxel 25 mg m^−2^ over 60 min on days 1, 8 and 15 of a 28-day cycle.

After two cycles of therapy, patients underwent follow-up CT scans for assessment of response according to the WHO criteria. Patients were seen every week in the outpatient clinic for laboratory tests and evaluation of toxicity and adverse events. Patients were treated until best response or until there was evidence of progression of disease or increasing side effects. Toxicity was graded using the NCI common toxicity criteria (version 2.0).

### Statistical considerations

The primary end point of the study was to determine the proportion of patients who respond to irinotecan plus docetaxel. The study was designed as a two-stage trial according to Gehan (1961) assuming a response rate of 15%. With a power of 90%, this resulted in a sample size of 15 for the first stage. The size of the second stage was determined by the observed number of responses and by the prespecified precision of 10%. All eligible patients were included in response, toxicity and survival analyses.

## RESULTS

### Experiences with irinotecan plus docetaxel once every 3 weeks

Four male patients (median age 55 years, range 53–65 years; ECOG performance status=one in all four patients, pretreated with cisplatinum-based combination chemotherapy in three patients and by cisplatinum-based chemoradiotherapy in one patient) were treated with irinotecan plus docetaxel once every 3 weeks. After four cycles administered in two patients and six cycles given in two patients, two patients achieved a partial remission and two patients had stable disease. All patients experienced febrile neutropenia at least once. Neutropenia grade 3/4 was noted in all 18 cycles that were administered. Other grade 3/4 toxicities were thrombocytopenia in one patient, anaemia in two patients, hyponatraemia due to diarrhoea in one patient, hypoglycaemia in one patient, nausea in one patient and asthenia in one patient. Toxicity necessitated dose reductions and/or delay of therapy in all patients. Mean irinotecan dose intensity (DI) of 45.4 mg m^−2^ week^−1^ was 85.2% of the planned DI; mean docetaxel DI of 17.5 mg m^−2^ week^−1^ was 80.6% of the planned DI. In order to reduce the haematological toxicity, the chemotherapy regimen was amended.

### Patient characteristics, response and survival with irinotecan and docetaxel 3 out of 4 weeks

After amendment of the study, from March 2001 to July 2002, 24 patients were enrolled. Patient characteristics are listed in Table 1

**Table 1:** Patient characteristics

**Characteristics**	
Total number of patients	*N*=24
Median age	58.5 (40–75) years
*Gender*	
Male	18
Female	6
*ECOG performance status*	
PS 0	9
PS 1	11
PS 2	4
*Histology*	
Adenocarcinoma	13
Epidermoid carcinoma	11
*Previous treatment*	
Chemotherapy	15
Chemoradiotherapy	5
Chemotherapy and chemoradiotherapy	4
*Sites of measurable disease*	
Lymph nodes	5
Lung	2
Liver	1
Oesophagus and lymph nodes	2
Oesophagus and lung	3
Oesophagus and liver	1
Lymph nodes and lung	3
Lymph nodes and bone	1
Lung and liver	1
Oesophagus and lymph nodes and liver	1
Oesophagus and lymph nodes and lung	1
Lymph nodes and liver and soft tissue	1
Oesophagus and lymph nodes and lung and liver	2

. Nine (37.5%) of the patients had undergone oesophagectomy, all patients had received cisplatin-based chemotherapy or chemoradiotherapy or both. Lymph nodes, lung and liver were the predominant sites of metastatic disease. In 11 patients (45.8%) tumours had not responded on first-line chemotherapy or chemoradiotherapy, whereas in 13 patients (54.2%) tumours had relapsed or progressed after initial complete or partial remission.

The patients completed a median of two cycles (range 0–4 cycles). In four patients, treatment was continued until best response. In 14 patients, treatment was discontinued due to disease progression. In six patients, adverse events or other medical complications impeded further treatment.

In 20 patients, tumour response was evaluable according to the WHO criteria. Four patients who did not complete two cycles were evaluated as having progressive disease. There were no complete responses. Three patients achieved a partial remission. The response rate was 12.5% (95% CI=2.7–32.4%). Overall, eight patients (33.3%, 95% CI=15.6–55.3%) had disease stabilisation (partial remission or stable disease) with a median duration of 18.5 weeks (range 16–51). Interestingly, those patients who achieved partial remission had a limited tumour burden with no more than two organs involved (one patient with nodal involvement, one patient with pulmonary involvement, one patient with nodal and pulmonary involvement). In contrast, those patients with more than two organs involved all experienced rapid disease progression. Of note, the three responders on irinotecan plus docetaxel had not received more than one pretreatment (one patient pretreated with chemotherapy, two patients pretreated with chemoradiotherapy). In contrast, in the six patients with more than one pretreatment (two patients after two chemotherapies, four patients after chemotherapy and chemoradiotherapy), five patients primarily progressed during treatment with irinotecan plus docetaxel and only one patient achieved disease stabilisation, which was of short duration (16 weeks). Response and disease stabilisation was equally associated with the two histological subtypes of oesophageal carcinoma.

**Figure 1:**
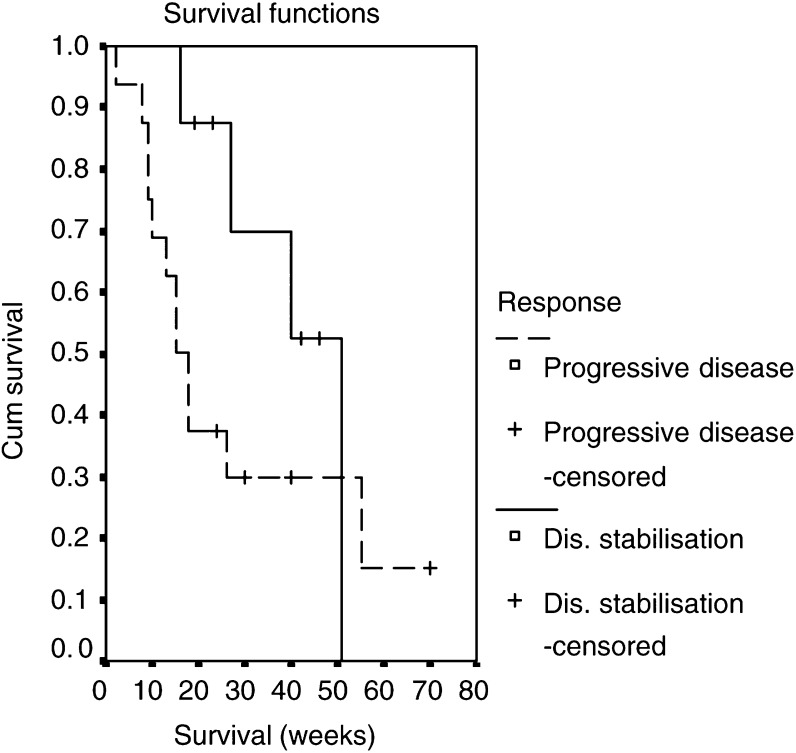
Kaplan–Meier survival for patients with progressive disease during chemotherapy (*n*=16) compared to those who achieved disease stabilisation (stable disease or partial response, *n*=8).

The median survival time was 26 weeks (range 2–70). In patients achieving disease stabilisation (partial remission or stable disease), median survival (51 weeks) was more than thrice as high compared to patients with progressive disease during chemotherapy (15 weeks; *P*=0.183, log-rank test). The Kaplan–Meier survival curves are shown in Figure 1

. The median progression-free survival was 9 weeks (range 2–51). To date, eight of the 24 patients (33.3%) are alive with a follow-up of 21 months.

### Toxicity with weekly irinotecan and docetaxel

After amendment of the study, the grade 3 and 4 toxicities included neutropenia, asthenia, diarrhoea, nausea/emesis and constipation (Table 2

**Table 2:** Toxicity of weekly irinotecan plus docetaxel chemotherapy

	**Grade 3/4**
**Toxicity**	***N* (%)**
Neutropenia	2 (8.3%)
Asthenia	5 (20.8%)
Diarrhoea	3 (12.5%)
Nausea/emesis	2 (8.3%)
Constipation	1 (4.2%)

*N*=Number of patients.

). Toxicity did not necessitate any dose reductions. Serious adverse events occurred in five patients, one with lethal outcome due to pneumonia. In contrast to the initial schedule, no patient experienced febrile neutropenia with weekly irinotecan plus docetaxel.

## DISCUSSION

The object of this study was to assess the activity and toxicity of irinotecan plus docetaxel in cisplatin-pretreated relapsed or refractory oesophageal cancer. Irinotecan plus docetaxel administered once every 3 weeks revealed unacceptable haematological toxicity. Therefore, this regimen had to be amended. With weekly irinotecan and docetaxel, which proved to be feasible, only 12.5% of the patients achieved major remissions, but the study was designed to obtain a response rate of at least 15.0%. Thus, at first glance, this study yielded negative results regarding the end points activity and toxicity. However, both end points deserve closer attention.

The combination of irinotecan plus docetaxel administered once every 3 weeks was established by two phase I studies published recently (Adjei *et al*, 2000; Couteau *et al*, 2000). Both studies enrolled patients with incurable cancer who had undergone at least one prior chemotherapy or radiotherapy. In both phase I studies, myelosuppression proved to be dose limiting. Therefore, not surprisingly the four patients treated before the amendment of this phase II study experienced neutropenia grade 3 or 4 with every cycle. However, in contrast to the data from the phase I studies indicating that febrile neutropenia occurred with a relatively low incidence of 14 and 22.5%, respectively (Adjei *et al*, 2000; Couteau *et al*, 2000), all four patients enrolled in this study required hospitalisation due to febrile neutropenia at least once during their treatment. The reasons for this unexpectedly high incidence rate of severe haematological toxicity are not evident. Patients were not older and their performance status was not worse compared to the patients included into the two published phase I studies. However, pretreatment of the oesophageal cancer patients enrolled into this phase II trial was probably more myelotoxic compared to Adjei's study that included even some patients without prior chemotherapy. Moreover, in our study no prophylactic growth factors were allowed, which in contrast were administered in the two published phase I studies. After this high complication rate became evident, the protocol committee decided to amend the protocol. The amendment took into account the dose intensity reached with 3-weekly irinotecan plus docetaxel as well as previously published data indicating a more favourable haematological toxicity profile, if both docetaxel and irinotecan were given in weekly schedules (Hainsworth *et al*, 1998; Murren *et al*, 2001). Indeed, with the administration of irinotecan plus docetaxel 3 out of 4 weeks, a dramatic decrease in myelosuppression was noted in comparison to the initial schedule. No more febrile neutropenia occurred. On the other hand, nonhaematological toxicity remained considerable. Asthenia was the predominant side effect. This finding corresponds with published data on protocols based on weekly infusions of docetaxel (Hainsworth *et al*, 1998).

With an objective response rate of 12.5%, the activity of weekly irinotecan plus docetaxel was lower than the 15.0% response rate envisaged in this study. However, this result correlates well with previous chemotherapy trials that have reported response rates of 6 and 0%, respectively, in patients with pretreated oesophageal cancer (Conroy *et al*, 1996, Heath *et al*, 2002) and clearly indicates the small likelihood of major remissions in cisplatin-pretreated oesophageal cancer patients. Therefore, beside the response rate one should also consider the rate of disease stabilisation indicating a potential value of a treatment regimen within a phase II study. With a rate of disease stabilisation of 33.3% (partial remissions and stable diseases) and a median duration of 33.5 weeks (16–51 weeks), one can assume that this subset of patients derived some benefit from this palliative treatment. This assumption is underlined by the notably longer median survival observed in the patients with disease stabilisation compared to patients with refractory disease. Of note, patients with a lower tumour burden had a higher chance to achieve a partial remission, whereas those patients with more than two organs involved all experienced early disease progression. Although the small number of patients does not justify any definite conclusions, this finding might influence careful decision making for second-line treatment in oesophageal cancer patients. Overall survival of the patients enrolled in this study was short with a median duration of 26 weeks. However, this was more than the median survival of 3.4 months reported in oesophageal cancer patients treated with single-agent docetaxel (Heath *et al*, 2002).

In conclusion, irinotecan plus docetaxel administered once every 3 weeks as recommended previously revealed excessive haematological toxicity and proved to be not feasible in the studied patient population. In contrast, with the regimen of irinotecan plus docetaxel 3 out of 4 weeks haematological toxicity was rather low. Nonhaematological toxicity remained considerable but manageable. The activity of weekly irinotecan plus docetaxel was moderate in extensively pretreated oesophageal cancer patients. Nevertheless, the response and survival data were better than the ones seen in previous single-agent chemotherapy trials and the results suggest a palliative benefit for the subset of patients who achieve disease stabilisation. Therefore, the search for less toxic and more active combination chemotherapy regimens in pretreated oesophageal cancer remains justified within clinical trials.
